# Simultaneous Assessment of Homonymous and Heteronymous Monosynaptic Reflex Excitability in the Adult Rat

**DOI:** 10.1523/ENEURO.0227-18.2018

**Published:** 2018-10-16

**Authors:** Calvin C. Smith, Roger W. P. Kissane, Samit Chakrabarty

**Affiliations:** 1 Department of Neuromuscular Diseases, UCL Queen Square Institute of Neurology, University College London, London, United Kingdom, WC1N 3BG,; 2 School of Biomedical Sciences, Faculty of Biological Sciences, University of Leeds, Leeds, United Kingdom, LS2 9JT

**Keywords:** Monosynaptic reflex, Homonymous, Heteronymous, modulation, corticospinal, reticulospinal

## Abstract

In order to successfully perform motor tasks such as locomotion, the central nervous system must coordinate contractions of antagonistic and synergistic muscles across multiple joints. This coordination is largely dependent upon the function of proprioceptive afferents (PAs), which make monosynaptic connections with homonymous motoneurons. Homonymous pathways have been well studied in both health and disease but their collateral fibers projecting to heteronymous, synergistic muscles receive relatively less attention. This is surprising given that PA collaterals have significant effects on the excitability of heteronymous motoneurons, and that their synaptic terminal density is activity dependent. It is likely that the relative lack of literature is due to the lack of a preparation which allows synergistic heteronymous pathways to be assessed *in vivo*. Here, we describe a method to simultaneously evoke homonymous and heteronymous (synergistic) monosynaptic reflexes (MSRs) and study their modulation by descending pathways in adult rats. Through stimulation of the medial plantar nerve, we were able to produce an H reflex in the intrinsic foot (IF) muscles of the hind paw with a latency of 10.52 ± 3.8 ms. Increasing the stimulus intensity evoked a robust signal with a monosynaptic latency (11.32 ± 0.35 ms), recorded in the ipsilateral gastrocnemius (Gs). Our subsequent analyses suggest that Gs motoneurons were activated via heteronymous afferent collaterals from the medial plantar nerve. These reflexes could be evoked bilaterally and were modulated by conditioning stimuli to the cortex (Cx) and reticular formation. Interestingly, cortical stimulation was equally efficient at modulating both ipsilateral and contralateral reflexes, indicating that cortical modulation of lumbar sensory afferents lacks the laterality demonstrated by studies of cortical muscle activation. This technique represents a novel, relatively simple way to assess heteronymous afferent pathways in normal motor control as well as in models of motor disorders where adaptive and maladaptive plasticity of PAs and descending systems affects functional outcomes.

## Significance Statement

This study describes a method for simultaneously recording bilateral homonymous and heteronymous PA reflexes in rodents. Bilateral stimulation of the medial plantar nerve produced homonymous H reflexes in IF muscles of both hind paws as well as heteronymous MSR in the Gs muscles. We used this technique to assess cortical and reticular modulation of homonymous and heteronymous reflexes. Surprisingly, cortical conditioning of reflexes revealed a lack of laterality in modulation of homonymous and heteronymous reflexes. This technique will be extremely useful for further studies of heteronymous afferent pathways in normal and dysfunctional motor states, such as spinal cord injury, stroke, and cerebral palsy.

## Introduction

To successfully perform motor tasks such as locomotion, the CNS must coordinate contractions of antagonistic and synergistic muscles across multiple joints. Previous work demonstrates that sensory information conveyed by peripheral receptors and their afferent fibers are essential to ensuring precise movement control (Pearson, 1995). The importance of sensory information to movement control increases following lesions to the descending control systems, in conditions such as spinal cord injury and cerebral palsy (Takeoka et al., 2014). While sensory afferents have been shown to be crucial to recovery of locomotor function, they are also implicated in development of dysfunctional states such as spasticity (Tan et al., 2012; Smith et al., 2017). So far, our understanding of these outcomes is based primarily on assessment of PA connections (and their modulation) between muscle spindles and motoneurons of the same (homonymous) muscle. However, PAs are known to make many projections to other, mainly synergistic motor pools (Eccles et al., 1957; Nelson and Mendell, 1978). These heteronymous connections are thought to be extremely important for establishing and coordinating muscle synergies to ensure biomechanical efficiency and stability of limb trajectories (Burkholder and Nicols, 2000). Despite this knowledge, synergistic PA collaterals are rarely studied in either normal or dysfunctional motor control. Considering the aberrant recruitment of muscle synergies in spastic and dystonic conditions, which is often sensory induced, the plasticity of these heteronymous connections and their modulation may be important mechanisms underlying motor dysfunction (Nielsen et al., 2007). This hypothesis is supported by the fact that PA terminal density on heteronymous, synergistic motoneurons is activity dependent (Mendelsohn et al., 2015).

The development of new techniques is crucial for furthering our understanding of neurophysiology in health and disease. The relative lack of studies assessing PA collateral projections to heteronymous motoneurons likely reflects the absence of a preparation permitting electrophysiological access to the circuit *in vivo*. Here, we describe a method by which stimulating the medial plantar nerves simultaneously evokes homonymous MSRs in the IF muscles and heteronymous MSRs from the Gs muscle. We characterize these reflexes in terms of their recruitment and paired pulse interaction profiles. Additionally, we assessed descending modulation of these reflexes by delivering conditioning stimuli to either the motor cortex or medial reticular formation (MRF).

The work describes a novel, relatively simple technique for assessing plasticity of homonymous and heteronymous afferents and their modulation by descending systems. These methods will be important for future study of sensory control of movement, especially in movement disorders such as spinal cord injury.

## Materials and Methods

Experiments and procedures were performed in a manner that conformed to the United Kingdom Animals (Scientific Procedures) Act 1986. Approval was granted by the local Animal Welfare and Ethics committee (University of Leeds).

### Animals

Male Wistar rats (339 ± 21 g) were used in all experiments. Number of animals for each experiment is indicated in Results.

### Preparation

Initial anesthesia was induced by intraperitoneal administration of a ketamine (100 mg/kg) and xylazine (5 mg/kg) mix, and the anesthetic plain maintained through intramuscular injections of ketamine (50 mg/kg) every 20–30 min. The animal was continuously checked for nociceptive reflexes and additional anesthetic administered when required. On loss of paw withdrawal, the hind limbs and top of the head were shaved. The animal was placed prone in a custom stereotaxic frame and the head was fixed in place with ear bars and mouthpiece. A heating pad maintained the animal temperature at 37°C.

### Simultaneous stimulation and recording of homonymous Intrinsic foot H reflex and heteronymous gastrocnemius MSR

**Figure 1.**
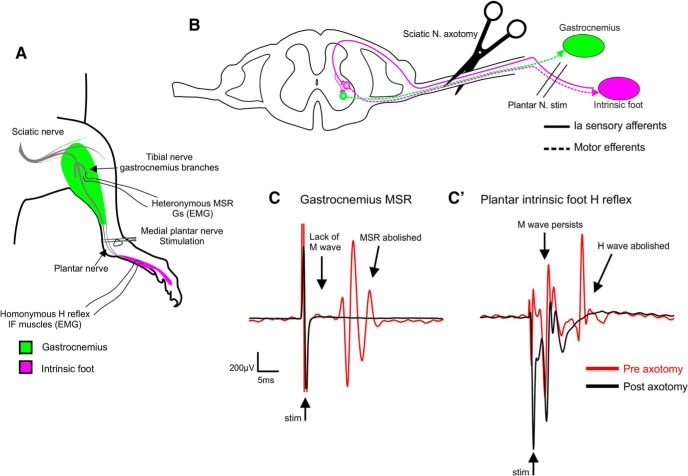
Experimental procedure for evoking homonymous H reflex and heteronymous MSR in IF and Gs muscles respectively. ***A***, Bipolar stimulation needles inserted at the medial maleolus, adjacent to the medial plantar nerve. Bipolar recording wire electrodes are inserted into the Gs and IF muscles. ***B***, Graphical representation of homonymous/heteronymous pathways and sciatic nerve transection for confirmation of synaptic signal. Solid lines represent monosynaptic PA pathway with collateral from IF PA to Gs motoneuron. Dashed lines represent motor efferents. ***C***, ***C’***, Traces showing Gs heteronymous MSR and homonymous IF H reflex before (red line) and after (black line) sciatic nerve axotomy. Notice that both late events are lost following axotomy suggesting they invole a synaptic component, whereas the early “M wave” remains. Double lines at the end of a stimulus artifact represent truncation for visual purposes.

Bilateral, subcutaneous bipolar needle electrodes (SpesMedica) were inserted adjacent to medial malleolus to stimulate medial plantar divisions of the tibial nerve. A pair of insulated fine copper wires (40 AWG; 79 µm in diameter) with 1.5- to 2-mm bared tips were inserted bilaterally into plantar IF muscles using a hypodermic needle (27 G). These wires recorded the homonymous H reflex. A pair of multi-stranded stainless-steel wires (Cooner), bared by 1.5–2 mm near the tip, were inserted bilaterally into the gastrocnemius/soleus muscles. These wires recorded the heteronymous MSR. The hind limbs rested naturally on the heating pad, with the ankle angle at ∼100°. To evoke homonymous H reflexes, square pulse current (0.3 ms) was delivered from a stimulator (ISO-STIM 01M Stimulus Isolator Module, npi electronic) driven by a Master-8 (A.M.P.I.) every 5 s. Thresholds were identified as the stimulation intensity at which the smallest visible response occurred at a frequency of 50%. Both IF H reflexes and Gs MSR were recorded simultaneously during graded stimulation of the medial plantar nerve until maximal IF M wave and Gs MSR were recorded (to produce a recruitment curve). Frequency-dependent depression/facilitation of the IF H reflex/Gs MSR was assessed by delivering paired pulses to the medial plantar nerve at intervals ranging from 1000 to 20 ms. For all conditioning experiments, IF H reflexes and Gs MSR were evoked on the ascending portion of their recruitment curves, as recorded at the beginning of each experiment (1.0–1.5 × threshold). This ensured that both facilitation and inhibition are possible (Crone et al., 1990; Knikou, 2008). Control (single stimulus) trials were performed immediately before test (double stimuli) for each time interval. This enabled us to express the test amplitudes as a percentage of the control and account for any changes in baseline MSR amplitudes occurring over time. At the end of some experiments, the sciatic nerve was transected proximal to the stimulation site. The late wave was confirmed unequivocally as synaptic (H wave or MSR) if it was abolished after axotomy (Fig. 1*C*,*C’*). The early M wave always remained post transection as this signal results from direct motor axon conduction.

### Cortical and reticular conditioning stimuli

Once the head was fixed in the stereotaxic frame using ear bars and mouthpiece, a midline incision was made on the skin covering the top of the skull. The skin was retracted and fascia removed from the skull. A 5 × 5 mm area of the skull over the motor cortex was thinned using a drill and the skull flap removed using forceps. The dura was then incised and retracted from the window. Petroleum jelly was used to build a wall around the skull window so that warmed paraffin oil could bath the exposed cortex, preventing desiccation. Using a micromanipulator (Narishige), a stainless steel microelectrode (Microprobes) was lowered 1.5 mm into the ankle flexor region of the motor cortex (Neafsey et al., 1986). Monopolar stimulation was performed between the microelectrode and a ground clip attached to the skin of the scalp. Square wave pulses with a duration of 0.3 ms were delivered at a frequency of 300 Hz. Five pulses were used to evoke a cortical motor-evoked potential (MEP) in the gastrocnemius muscle, at which point the stimulation was reduced to sub-motor threshold. Only three pulses were used for conditioning stimuli. If triceps surae were not the primary muscle group activated, the position of the cortical electrode was adjusted. Sub-threshold stimuli were defined as maximum intensities at which no MEP was observed after 10 consecutive trials separated by 5 s. Cortical conditioning stimuli were delivered 20 ms before medial plantar nerve stimulation as this was the latency to cortically evoked MEPs. Control IF reflexes were recorded, followed by cortical conditioning of IF MSRs; this was completed for both contralateral and ipsilateral sides by simply switching the stimulation to the contralateral plantar nerve. For conditioning experiments, IF H reflexes and Gs MSRs were evoked on the ascending portion of their recruitment curve so that both facilitation and inhibition were possible. Although IF H reflexes were also present during Gs MSRs, these responses were not quantified, as they were likely to be on the descending portion of their recruitment curve.

Coordinates from the Paxinos rat atlas were used to stimulate the MRF; however, stimulation sites were used for sub-threshold conditioning if they elicited a clear hindlimb response. Motor responses to MRF stimulation varied. We saw activation of all four limbs, both hindlimbs only, both forelimbs only, or a combination of hindlimbs and forelimbs. Due to variations in anatomic landmarks between animals, it was not possible to reproduce outputs between animals when specific coordinates were used. Therefore, a range of coordinates were used. In the rostro-caudal axis, stimulation sites were between 1 and 3.5 mm caudal to the interaural line (IA). The medio-lateral range was 1–2.5 mm lateral from midline. The depth of the tip of the electrode was 7–9 mm below the dura. We predict that most of the conditioning stimulation sites were within the gigantocellular nucleus of the reticular formation, however at depths of 7–8 mm, the MLF was also likely to be activated.

### Analysis

All signals were recorded with a preamplifier (Digitimer) and sampled with Signal 5 (CED) software. Quantification of reflex amplitudes were made from averages of 15–30 traces which were generated using Signal 5. Depression of the MSR in response to homonymous paired pulse stimuli was calculated by expressing the test reflex as a percentage of control reflex. Averaged reflexes evoked after a conditioning stimulus to either cortex or reticular formation were compared to control trials which the reflex was evoked with no conditioning. A reflex was facilitated if the average amplitude was >10% compared to control, or if the threshold of the response was reduced (Lundberg et al., 1962). The threshold was lowered if the addition of conditioning stimuli resulted in consistent reflex responses that were not observed in the absence of conditioning (sub-threshold afferent stimulation). A reflex response was inhibited if its amplitude was reduced by 10% or greater compared to the control average. All values are expressed as SEM.

## Results

### Medial plantar nerve stimulation evokes homonymous H reflex in Intrinsic foot muscles and heteronymous MSR in gastrocnemius via afferent collaterals

To simultaneously evoke homonymous IF muscle and heteronymous Gs MSRs, we stimulated the medial plantar nerve and recorded from both IF and Gs muscles (Fig. 1*A*,*B*). There were two signals detected in the IF muscles. The first (M wave) occurred at 2.84 ± 0.16 ms and the second (H wave) occurred at 10.52 ± 3.8 ms (*n* = 12), representing the two major components of the H reflex (Fig. 1*C’* ; Meinck, 1976). In the Gs muscles, there was a solitary late signal at 11.32 ± 0.35 ms (*n* = 10; Fig. 1*C*). There could have been several explanations for the stimulus evoked signal detected during simultaneous EMG recording from Gs. First, because the medial plantar nerve is a branch of the tibial nerve, which innervates Gs muscle proximally, we wanted to rule out electrotonic spread of current to Gs motor and afferent fibers in the tibial nerve. Gs motor nerve activation (M wave) was ruled out because the latency was too long (IF M wave = 2.84 ± 0.16, *n* = 12 versus Gs response = 11.32 ± 0.35 ms, *n* = 10), and the response was abolished on transection of the sciatic nerve, suggesting a synaptic pathway (Fig. 1*B*). A synaptic response could result from either activation of Gs afferent fibers in the tibial nerve or heteronymous intraspinal collaterals from the plantar nerve projecting to Gs motoneurones (Eccles et al., 1957). The latency of the Gs response was 1.18 ± 0.12 ms later than the IF H reflex and the Gs threshold (20.12 ± 3.88 µA) was 2.38 ± 0.36 times greater than IF H reflex threshold (8.63 ± 1.20 µA). If the Gs response resulted from activation of homonymous Gs afferents, the latency would be earlier than the more distal IF muscle. Moreover, increasing stimulation intensity would eventually result in activation of motor fibers in the tibial nerve, yet we never observed an early M response in the Gs muscle, even at stimulation intensities of 100 µA (Fig. 1*C*).

### Comparing intrinsic foot H reflex to heteronymous gastrocnemius MSR

#### Recruitment curves

**Figure 2.**
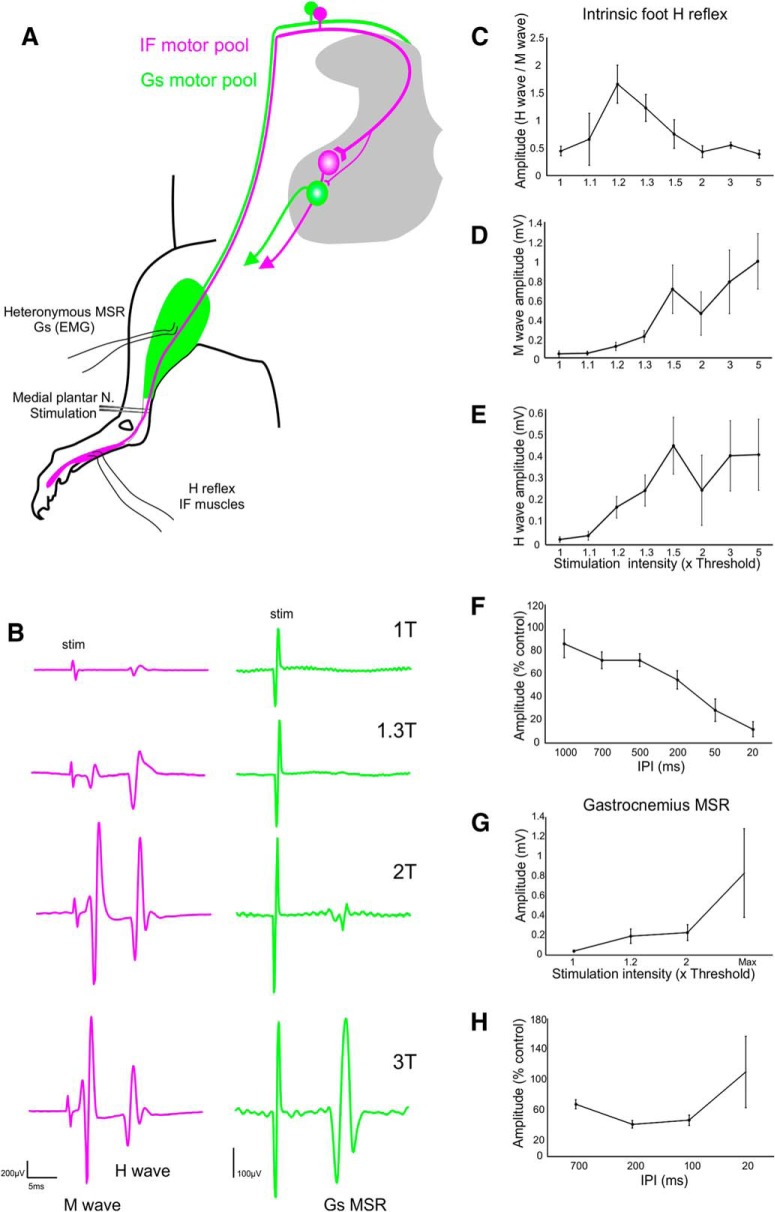
Characterization of homonymous IF H reflex alongside heteronymous Gs MSR. ***A***, Schematic of experimental set up. ***B***, Example of simultaneous EMG recording from IF (left) and Gs (right) during graded stimulation of the medial plantar nerve. ***C–F,*** Recruitment characteristics of the H reflex and paired pulse interactions. ***G–H***, Heteronymous Gs recruitment curve and paired pulse interactions.

We performed graded stimulation of the medial plantar nerve to establish recruitment profiles for both the H reflex and heteronymous Gs MSR. For the H reflex recruitment curve, the H wave was expressed as a ratio of the M wave and showed a classical profile (Knikou, 2008). In contrast to the H wave, the heteronymous Gs MSR increased with stimulation intensity up to its maximum value and was not reduced by further increases (Fig. 2*C–H*). This is due to a lack of M wave for the heteronymous reflex, meaning the synaptic response is not subject to antidromic collisions.

#### Paired pulse interactions

In response to paired stimuli, the H reflex classically shows frequency-dependent depression, which becomes complete at high frequencies (Ho and Waite, 2002; Tan et al., 2012; Kathe et al., 2016). The depression seen at lower frequency paired stimuli is thought to be due to both inhibitory mechanisms as well availability of neurotransmitter following the previous Ia terminal activation, and is therefore an important assay for assessing synaptic efficacy and modulation of afferent fibers (Hultborn et al., 1996; Mende et al., 2016; Smith et al., 2017).

To further characterize the heteronymous Gs reflex, we compared the responses to conditioning pulses to the same nerve at several different time intervals. In agreement with previous literature, the IF H reflex experienced homosynaptic depression with increasing magnitude as stimulation interval times were reduced. For example, at 200-ms depression was 54.88 ± 7.9% (*n* = 6), and at 20 ms, depression was complete in some animals with the mean value being 11.97 ± 6.59% (*n* = 5). For the Gs heteronymous reflex, the test response was 42.08 ± 4.95% at 200 ms (*n* = 5) but 110.43 ± 46.59% at 20 ms (*n* = 5). This suggests that, in contrast to the H reflex, the heteronymous Gs reflex shows depression at lower stimulation frequencies but is more likely to be facilitated at higher frequencies. This is consistent with the work of Eccles and Rall (1951), who showed that paired homonymous stimulation resulted in reflex facilitation at very short intervals and depression at longer stimulus intervals. Early facilitation of the heteronymous reflex likely reflects a summation of stimuli at afferent collateral branch points, thereby reducing transmission failures (Wall and McMahon, 1994).

### Cortical conditioning stimuli exhibit distinct modulation of homonymous and heteronymous MSR

**Figure 3.**
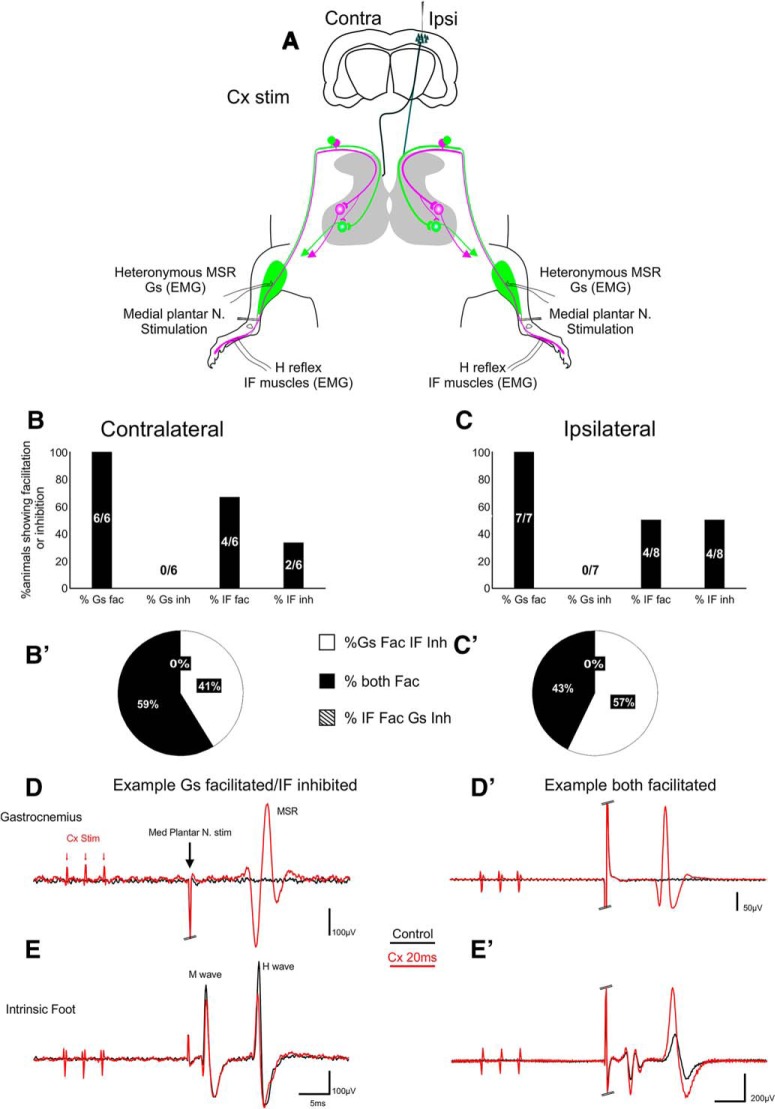
Cortical modulation of homo/heteronymous reflexes bilaterally. ***A***, Schematic of the experimental set up. Cortex was stimulated ipsilaterally and conditioning pulses preceded either ipsi- or contralateral medial plantar nerve stimulation by 20 ms. ***B***, ***C***, Percentage of animals in which Cx conditioning stimuli induced facilitation of both Gs and IF reflexes or facilitated Gs but inhibited IF. ***D***, ***E***, Example of animal in which Gs is facilitated and IF inhibited. ***D’***, ***E’***, Example of animal in which both Gs and IF reflexes are facilitated. Double lines at the end of a stimulus artifact represent truncation for visual purposes.

Next, we determined the effects of cortical conditioning stimuli on the amplitude of the MSR. To do this, we used sub-threshold cortical stimulation to ensure that both inhibition and facilitation of the MSR were possible (Fig. 3*A*). Supra-threshold stimulation would result in cortical MEPs, which are likely to mask the effects of interposed inhibitory pathways to motoneurons.

Cortical stimuli preceding plantar nerve stimulation by 20 ms resulted in either facilitation or inhibition of the IF muscle H reflex (Fig. 3*B–E’* ). Facilitation was seen both when IF H wave was supra- and sub-threshold. Interestingly, we observed no obvious laterality for Cx facilitation of IF H reflex (contralateral facilitation = 4/6 rats, ipsilateral facilitation = 4/8 rats) but inhibition was less likely for the contralateral (2/6 rats) compared to the ipsilateral CST (4/8 rats; Fig. 3*B*,*C*). It is widely appreciated that the CST is mainly a crossed pathway and that MEPs are rarely evoked ipsilaterally (Neafsey et al., 1986). Indeed, in our preparation, supra-threshold cortical stimulation only produced MEPs in the contralateral limbs. Surprisingly however, we found that cortical stimulation always resulted in bilateral modulation of both homonymous and heteronymous MSRs. Moreover, we found that 100% of heteronymous Gs MSRs were strongly facilitated by ipsilateral and contralateral cortical stimulation. This included both threshold reduction at sub-threshold stimulus intensities, and increased response amplitude at supra-threshold intensities. These results suggest a lack of laterality for cortical modulation of lumbar proprioceptive reflexes.

### MRF stimulation differentially modulates ipsi- and contralateral intrinsic foot muscle H reflexes

**Figure 4.**
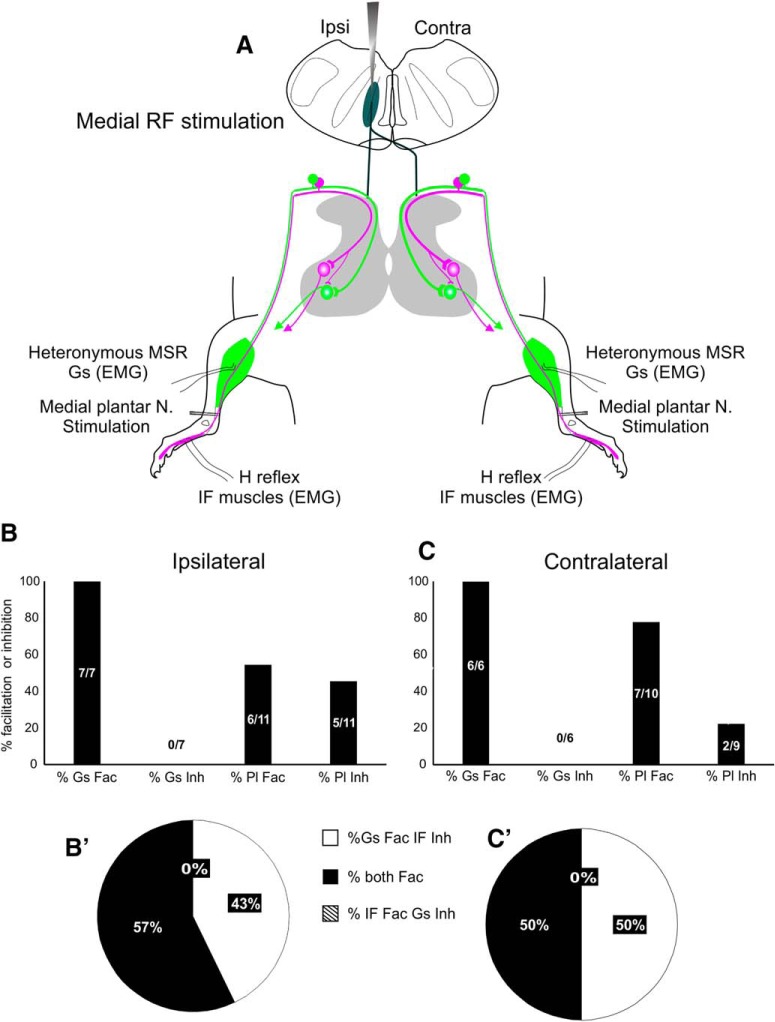
Modulation of bilateral homonymous and heteronymous reflexes by stimulation of the MRF. ***A***, Schematic of the experimental set up. MRF was stimulated ipsilaterally and conditioning pulses preceded either ipsi- or contralateral medial plantar nerve stimulation by 20 ms. ***B***, ***C***, Percentage of animals in which MRF conditioning stimuli induced facilitation or inhibition of Gs and IF MSRs.

We were also able to test bulbospinal modulation of lumbar proprioceptive reflexes by administering conditioning stimuli within the MRF (Fig. 4*A*). MRF stimulation, also preceding MSRs by 20 ms, resulted in both inhibition and facilitation of the IF H reflex. For ipsilateral conditioning stimuli, either facilitation or inhibition of the H reflex was seen at a frequency of 50% (Fig. 4*B*,*C*). For contralateral conditioning stimuli, plantar H reflexes were more likely to be facilitated (5/7) than inhibited (2/7). Similar to cortical conditioning, heteronymous Gs MSRs were facilitated bilaterally in 100% of animals.

## Discussion

This study describes a relatively non-invasive protocol for recording bilateral reflexes across two different joints via simultaneous activation of homonymous IF muscle afferents and their heteronymous collaterals to Gs motoneurons. Modulation of these pathways can be assessed by conditioning stimuli to supraspinal structures such as the motor cortex and medullary reticular formation. Our results show that IF homonymous and Gs heteronymous reflexes are differentially modulated by supraspinal conditioning stimuli. IF H reflexes were modulated bidirectionally while Gs MSRs were facilitated only. Remarkably, we show that cortical modulation of these reflexes lacks the heavily weighted laterality predicted from previous anatomic and MEP physiologic studies.

Proficient control of movement involves the coordination of many synergistic and antagonistic muscles bilaterally. Much of our knowledge regarding sensory contributions to movement control in healthy and injured states come from the assessment of afferent input to single muscles. However, it is well appreciated that low threshold Ia PA make monosynaptic connections with their homonymous motoneurons as well as many other heteronymous, synergist motoneurons (Baldissera et al., 1981; Hultborn, 2006). This has been demonstrated in experiments using both electrical stimulation of peripheral nerves and stretch activation of muscle spindles (Eccles et al., 1957; Edgley et al., 1986; Wilmink and Nichols, 2003). Heteronymous afferent connections are important for establishing and coordinating muscle synergies in motor control, but are rarely assessed in normal or dysfunctional states. Recent data have reinforced the importance for greater understanding of afferent plasticity in health and disease, beyond that of direct homonymous connections. For example, Mendelsohn et al. (2015) used a clever genetic strategy to show that that the formation of heteronymous afferent terminals on synergist MNs is activity dependent. Similarly, establishment of normal PA input to Renshaw cells (presumably from collateral branches) is dependent on activity from descending systems during postnatal development (Smith et al., 2017). Despite the clear need for development of such knowledge, the lack of a well-defined technique for routinely assessing heteronymous afferent systems has proved a significant barrier to progression.

This study overcomes that barrier by describing a method to record heteronymous, synergistic MSRs in the Gs while recording H reflexes in the IF muscles. It therefore confers a major advancement in the capability to investigate mechanisms contributing to motor dysfunction and recovery in the spinal cord. First, the protocol allows recording of MSRs bilaterally, which is significant considering the bilateral nature of mammalian motor control, especially locomotion. Second, simultaneously recording heteronymous MSRs across different joints permits investigation of muscle synergy interactions in the healthy spinal cord, disease or injury states, and after therapy. Finally, efficient interaction with the environment necessitates functionality in both gross and fine motor tasks. Specific training is needed to promote recovery of such tasks and often training of one task can be detrimental to the recovery (negative transfer) of another (De Leon et al., 1999; Marsh et al., 2011). Our method allows afferent control of motor output to be assessed at proximal muscles involved in gross tasks such as locomotion as well as smaller distal muscles, which are associated with skilled tasks such as object manipulation and climbing. We propose that future studies assessing plasticity of PA following lesions to descending systems and subsequent therapies, should use bilateral, multi-joint assessments of PA function to provide further insight into potential mechanisms of motor dysfunction and recovery.

Interestingly, we show that cortical and MRF conditioning stimuli always facilitated bilateral Gs MSRs, but homonymous IF foot H reflexes could be either inhibited or facilitated. This makes sense functionally, as greater supraspinal control over the smaller distal musculature of the hind paws, which control digit movements, may be more advantageous for tasks such as climbing and object manipulation compared to the mainly locomotor function of the gastrocnemius muscles.

Facilitation of reflexes could occur via several mechanisms. First, PA terminals are subject to presynaptic inhibition (PSI) via GABApre interneurons, which receive both excitatory and inhibitory modulation from supraspinal systems (Rudomin and Schmidt, 1999). It has previously been demonstrated that cortical conditioning stimuli facilitate H reflexes in the hindlimb of humans via reduced PSI; therefore, disinhibition of tonic GABApre-mediated PSI may be responsible for facilitation of the MSR (Iles, 1996). Similarly, opposing effects of supraspinal stimulation on both reflexes might be due to increased PSI of IF PA in conjunction with increased excitatory drive to Gs. As rodents lack direct cortico-motoneuronal connections, the CST innervates intermediate excitatory pre-motor INs in the spinal cord which are responsible for cortical evoked MEP responses. (Ueno et al., 2018). It is possible that facilitation of Gs MSR may occur via summation of PA and CST excitation of the intermediate INs involved in eliciting cortical MEPs.

We hypothesize that the reason Gs heteronymous MSRs were always facilitated by conditioning stimuli to Cx and MRF is related to a reduction in branch point failures in IF heteronymous collateral fibers. These failures in action potential transmission are common, especially in long intersegmental branch projections such as those from IF afferents (mainly L5) to Gs MNs (L4–L5; Nicolopoulos‐Stournaras and Iles, 1983; Crockett et al., 1987; Wall and McMahon, 1994). Li et al. (2017) suggested that primary afferent depolarization (PAD), mediated by presynaptic GABApre terminals is able to bring collateral afferents closer to threshold, therefore increasing the probability of AP propagation through presynaptic facilitation (PSF). In agreement, we propose that GABA-mediated PAD acts to produce the classical inhibitory shunt of action potentials in homonymous IF PA but brings branching Gs heteronymous collaterals closer to threshold for AP generation, meaning that IF H reflexes are inhibited while Gs MSRs are facilitated. Although we suggest this as a likely mechanism, further studies are needed to confirm the hypothesis.

Our most surprising finding was that cortical conditioning of bilateral homonymous and heteronymous PA reflexes showed a lack of laterality in modulation. Although the mainly contralateral (5% ipsilateral, 95% contralateral) projecting CST is the most direct pathway, the cortex has diffuse and divergent projections to many subcortical and spinal nuclei which subsequently impinge on spinal motor and premotor neurons (Lemon, 2008). Our results support the idea that cortical modulation of spinal circuits is mediated by a “cortical motor system”, which includes a relatively small contribution from direct contralateral projecting CST axons compared to the many divergent subcortical projections. Indeed, it has been demonstrated across multiple species that corticospinal tract lesions have minimal effects on locomotor abilities (Lawrence and Kuypers, 1968; Muir and Whishaw, 1999). Although we cannot conclude which pathways mediate the ipsilateral cortical modulation of both heteronymous and homonymous MSRs, we speculate that cortico-bulbar pathways are utilized as bulbo-spinal projections lack strong laterality in rats (Armstrong, 1988). Additionally, the relatively fast conduction velocity of reticulospinal axons could account for synaptic delay in the reticular formation (Alstermark et al., 2004). The mechanism of facilitation of both MSRs would be different for bulbo-spinal projections because they synapse directly on MNs, and could therefore exert their effects by depolarizing the membrane potential, enabling a greater proportion of motor units to be recruited for a given stimulus, or the opposite. However, the bulbo-spinal tract could also use similar mechanisms to the CST such as PSI, PSF and other intermediate spinal premotor interneuron populations (Engberg et al., 1968; Rudomin, 1990; Alstermark et al., 2004).

Our results describe a novel, relatively non-invasive technique for assessing the physiology of homonymous and heteronymous afferent pathways in rats. This protocol will prove particularly useful to researchers aiming to further delineate mechanisms of motor dysfunction and recovery in disease and injury states, beyond what is currently possible with single muscle, homonymous H reflex assessments.

## Synthesis

Reviewing Editor: Robert Kalb, Northwestern University Decisions are customarily a result of the Reviewing Editor and the peer reviewers coming together and discussing their recommendations until a consensus is reached. When revisions are invited, a fact-based synthesis statement explaining their decision and outlining what is needed to prepare a revision will be listed below. The following reviewer(s) agreed to reveal their identity: Lea Ziskind-Conhaim.

Both reviewers felt that the work was interesting and useful. Their main criticisms focused on how the data was presented and discussed. While further experimentation is encouraged, it is not required. The most important point is that the authors follow the recommendations of the reviewers on data presentation and discussion

## Author Response

Author responses

Synthesis of Reviews:

Computational Neuroscience Model Code Accessibility Comments for Author (Required):

N/A

Significance Statement Comments for Author (Required):

The Significance statement was fine

Comments on the Visual Abstract for Author (Required):

Not applicable

Synthesis Statement for Author (Required):

Both reviewers felt that the work was interesting and useful. Their main criticisms focused on how the data was presented and discussed. While further experimentation is encouraged, it is not required. The most important point is that the authors follow the recommendations of the reviewers on data presentation and discussion

Reviewer #1

The manuscript entitled “Simultaneous assessment of homonymous and heteronymous monosynaptic reflex excitability in the adult rat" reports the development of a method to evoke homonymous and heteronymous proprioceptive-induced monosynaptic responses simultaneously and how these responses are modulated by stimulation of certain descending pathways. The study is pretty straight forward, with clear results. I only have a few points that need to be addressed prior to publication that I feel will aid the impact and clarity of the authors' interpretations.

1. The authors are strongly encouraged to present the actual amplitude of the H-reflex for the intrinsic foot muscle (in figure 2C); instead the authors present the H/M ratio. This is important because in figure 2D, the authors give the actual amplitude for the Gastrocnemius MSR. By providing the actual amplitude for the intrinsic foot H-reflex, the reader will be able to discern the relative contribution of the heteronymous proprioceptive fibers on Gastrocnemius. In addition, since this is a methodological paper, the reader will also have a better idea with respect to the actual values of the H-reflex from the intrinsic foot muscle.

We have added a graph with the H and M wave amplitudes as requested (Fig. 2D-E).

2. The authors are also encouraged to present the entire recordings including baseline and the stimulus artifact, whenever they present traces. In addition, I strongly encourage the authors to avoid using dotted lines for traces because it is difficult to delineate differences with respect to latency. Since the authors decided to use colors, they may opt for different colors when they superimpose traces (figs.1C and C'; figs.3D-E';

We agree with the reviewer and have changed all the traces to include stimulus artifacts and baselines. Dotted line traces have been replaced with Solid lines coloured black and red.

3. In the Analysis section (page 7), the authors state: “A reflex was facilitated if the amplitude of the averaged 155 was &gt;10% compared to control". Based on which evidence or previously published paper the authors used this criterion?

This was deemed to be an appropriate threshold to set as previous work assessing descending conditioning of MSRs showed modulation of test responses from 5-100% of the control {Lundberg, 1962}. We have added the reference to the text.

4. In figure 1C (Gastrocnemius MSR), the authors state that the latency of the response was 11.32ms, however, in the actual trace shown in figure 1C, the latency of the response appears to be 5 or 6ms. Why the response is so much shorter than the average latency?

The response latency may seem shorter than average by estimating by eye, but it is in fact within the stated SEM if the scale bar is used. As requested above, we have altered the way these traces are presented so that it is easier to delineate latencies (included stim artefacts).

5. The authors in the Abstract and Introduction state that the collateral proprioceptive fibers projecting to heteronymous and synergistic muscles is not well understood or studies. However, they failed to cite relevant papers. For instance, the authors should cite the following two previously published papers: i) Mendelsohn et al, 2015, Neuron and ii) Burkholder and Nicols, 2000, Motor Control.

We thank the reviewer for noticing this deficiency, and we altered the abstract and introduction accordingly (lines 8-12, page1, lines 57-69, page3).

Reviewer #2

The manuscript describes an innovative technique designed to evaluate the plasticity of homonymous and heteronymous proprioceptive afferent reflexes, and their regulation by cortical and reticular pathways. The medial plantar nerve was stimulated bilaterally and afferent responses were recorded simultaneously in the intrinsic foot (IF) muscles and the gastrocnemius muscles (Gs) of adult rats. This is the first study to examine: a) the plasticity of both homonymous and heteronymous reflexes simultaneously, and b) the role descending systems play in modulating their activities. The most intriguing, unexpected observation is that conditioning stimuli of the motor cortex and the medial reticular formation facilitates bilaterally the synergistic monosynaptic reflexes and facilitates or inhibits the H reflexes.

The manuscript is well-written with clear explanations and illustrations of the experimental procedures and the findings. It outlines a conceptually simple yet technically difficult method to assess proprioceptive plasticity and its regulation by descending pathways known to control movements. Most of the findings are new and quite interesting. What is missing is a detailed discussion of the data, suggesting possible cellular/network mechanisms and functional implications. Without understanding the factors involved in these modulatory responses it will be difficult to use this technique “for further studies of heteronymous afferent pathways in normal and dysfunctional motor states."

General and Specific Comments:

1.The manuscript does not include statistical analyses to confirm significance differences between data points.

We decided not to include statistical analyses in this paper because based on our measurements, we did not feel it added to the paper. For descending modulation of reflexes we set a threshold for significant modulation based on previous work and therefore our results were essentially binary. Eg facilitation/inhibition/no effect. Additionally, we do not make comparisons between groups and the homonymous IF H reflex and Gs MSR are not comparable as they are 2 different measures.

2.Determination of monosynaptic responses is based on synaptic delay, and the apparent frequency-dependent depression of the H reflex in the IF muscles (Fig. 2 E). Yet, unlike typical monosynaptic potentials, the latency of the recorded H reflexes is highly variable (10.52 {plus minus} 3.8 ms, SEM) compared to the monosynaptic latency recorded in the synergistic Gs muscles (11.32 {plus minus} 0.35 ms). Any explanation for the large variability?

The large variability is likely due to unavoidable variability in the positioning of both EMG and stimulation electrodes and nerve lengths in animals of different sizes (although we try to minimise this as much as possible). Additionally, it also likely reflects the relative complexity of the H reflex circuit. For example, there are collisions of anti and orthodromic signals in the motor nerve, which impact upon the stability of H reflex latency. Indeed, it is quite normal to see considerable variability in H reflex latency between consecutive stimulations.

This is not the case in the heteronymous Gs signal, which represents a more classical MSR involving afferent activation of motoneurons and an unimpeded conduction of orthodromic motor axon potentials to the muscle. Due to these reasons, H reflex latency is an unreliable metric (when comparing H reflexes between different animals in different recording sessions) unless normalised with an intra-animal control value. However, in our study the latency enables us to rule out activation of motor fibres and electrotonic spread of the stimulation to the muscle as a cause for the Gs signal as the latency would be much shorter in these cases.

3.Page 9, 1st Paragraph, and Fig. 2 B-C. The example shown in Fig. 2 B does not match the value presented in Fig. 2 C. At 2T stimulation the ratio H wave/M wave appears to be around 1 yet in Fig. 2 C the value is less than 0.5 with a small SEM.

The value of this example was 0.76 (H/M) which would be within the SD. For clarity, we have changed the traces to include a more representative example.

4.What was the stimulus intensity in pair pulse interactions? Was it at “maximal IF M wave and Gs MSR" as stated in Page 5, line 10? Stimulus intensity needs to be stated in each experimental paradigm.

We agree that more detail is needed here for clarity. For the paired pulse paradigms we always used a stimulation intensity which evoked H waves and Gs MSRs on the ascending portion of their recruitment curves (1-15*threshold) to ensure that both facilitation and inhibition were possible. More detail has been added to the methods section (lines 112-118)

It is important to discuss possible mechanisms that underlie the facilitation of Gs MSR at 20 ms pair stimuli. Was the plantar nerve stimulated at subthreshold intensity to produce a Gs MSR when only one stimulus was applied?

We have added a discussion point on this matter (232-237). Both stimuli were of the same intensity, which was supra threshold for Gs, but on the ascending portion of the recruitment curve so that both facilitation and inhibition were possible. This is now better explained in the methods. (lines 116-122)

5.Fig. 4: Conditional cortical and reticular formation stimuli always triggered bilateral facilitated Gs MSRs, but it either inhibited or facilitated IF H reflexes. It is desirable to discuss possible mechanisms and the functional significance of the different responses recorded in the homonymous and heteronymous muscles.

We have re-written our discussion to better discuss our findings.

6.At least two experiments come to mind that might explain some of the different responses recorded in the two muscles during conditional stimuli. a) Plantar nerve stimulation of &gt;2T should be used to produce Gs MSRs in controls. Inhibition cannot be detected if the plantar nerve stimulation does not produce Gs MSRs. b) It is important to determine whether the properties of Gs H reflexes generated by direct sciatic nerve stimulation (cut the tibial nerve) are similar to the MSRs generated plantar nerve stimuli.

a. We have done these experiments, but as stated in the text, conditioning pulses to the descending systems always facilitated the Gs MSR, whether supra or sub threshold. In the figures we show a reduction of the threshold for the Gs MSR with supraspinal stimulation as this is the clearest example of facilitation. This threshold reduction was always seen in conjunction with increased response amplitude when Gs was suprathreshold.

b. We thank the reviewer for the suggestion but we do not believe that these two techniques can be compared directly in the same muscle. H reflexes involve the stimulation of both motor and sensory fibres innervating the recorded muscle. Our heteromymous MSR is recruited via afferent collaterals within the spinal cord which are not subject to antidromic collisions. Therefore, the physiology involved in these reflexes is quite different. H reflexes evoked from the Gastrocnemius/soleus have been well characterised by other groups eg Smith et al 2017.
